# Complete Genome Sequence of Brucella canis GB1, a Strain Isolated from a Poodle in Beijing, China

**DOI:** 10.1128/MRA.01476-18

**Published:** 2019-01-10

**Authors:** Guanlong Xu, Rujia Cheng, Cuili Sun, Ge Zhang, Qiuchen Li, Shijing Sun, Hui Jiang, Junsheng Cheng, Yuming Qin, Liangquan Zhu, Jiabo Ding

**Affiliations:** a National Reference Laboratory for Animal Brucellosis, China Institute of Veterinary Drug Control, Beijing, China; b College of Veterinary Medicine, Shandong Agricultural University, Tai’an, Shandong, China; Queens College

## Abstract

Brucella spp. are facultative intracellular pathogens and zoonotic agents which pose a great threat to human health. Twelve different Brucella species have been identiﬁed to date. Here, we report the complete genome sequence of a Brucella canis GB1 strain, which contains two circular chromosomes of 3,277,308 bp in total.

## Abstract (precis)

Brucella spp. are facultative intracellular pathogens and zoonotic agents which pose a great threat to human health. Twelve different Brucella species have been identiﬁed to date.

## ANNOUNCEMENT

Brucellosis is an important chronic zoonotic infectious disease caused by bacteria of the genus Brucella. According to pathogenicity, phenotypic characteristics, and host preferences, Brucella spp. can be divided into 12 species; these include (with primary hosts) B. melitensis (sheep and goats), B. abortus (cattle), B. suis (swine), B. canis (dogs), B. ovis (sheep), B. neotomae (wood rats), B. ceti (dolphins, porpoises, and whales), B. pinnipedialis (seals), B. microti (voles and foxes), B. inopinata (humans), B. papionis (baboons), and B. vulpis (foxes) (1, 2). Among the 12 species of Brucella, B. abortus, B. melitensis, B. suis, and B. canis are pathogenic to humans. In 1966, B. canis was first isolated by Leland Carmichael from tissue samples from aborted beagle dogs (3). B. canis can cause miscarriages in livestock and infections in monkeys, rabbits, and humans, which have resulted in negative economic and public health impacts. Complete genome sequencing and genome analysis will help in unraveling the biological information of brucellosis, and this will ultimately aid in the prevention and control of B. canis. In this study, we report the genome sequence of B. canis GB1, which was isolated from an aborted poodle dog in Beijing, China.

The blood of an aborted poodle was cultured in the selective culture medium. A Brucella isolate was obtained and further identified by morphological observation, cultural and biochemical characterization, agglutination test, and AMOS-PCR analyses. Our results demonstrated that the isolate belonged to B. canis. Brucella DNA isolation was performed using TaKaRa MiniBEST bacterial genomic DNA extraction kit version 3.0 (catalog number 9763), according to the manufacturer’s protocol. Briefly, bacterial cell lysate was obtained using proteinase K and RNase A, and then genomic DNA was precipitated by adding 100% ethanol and collected in a spin column. After two washes, genomic DNA was eluted using the elution buffer from the kit. The DNA library was constructed using the TruSeq DNA library prep kit (Illumina, USA), according to the manufacturer’s instructions. In brief, DNA was fragmented by an ultrasonic method, and the end repairing of the fragments was conducted, followed by adaptor ligation. Then, the ligated DNA fragments ranging from 300 bp to 500 bp were purified, PCR amplified, normalized, and sequenced using the HiSeq 4000 platform with at least 100× coverage to obtain the raw data. The raw data were filtered using the FastQC software (version 11.5) to detect and remove the following low-quality sequence reads: (i) read length of <50 bp, and (ii) low-quality reads (>13% N, which was not identified as A/T/C/G). The redundant sequences or the reads with a primer/adaptor were also removed. SAMtools (version 1.3.1) (4) was used to determine the depth and coverage of the clean data. All low-quality bases (Q20 Phred quality score) from the sequencing reads were trimmed and then mapped to the genomic sequences to determine the optimum reference for B. canis BJ1 based on similarity. Consensus sequences from the reads mapped to the selected references were then generated by SAMtools. Unmapped reads were used as query sequences for BLASTN searches against the nucleotide (NT) database (using options “-e 1e-15 -F F”). Meanwhile, by comparing the sequencing depth and coverage with different genomes, the closed genomes were identiﬁed (5). The paired-end reads were assembled *de novo* using the Velvet 1.2.10 (6) software. The contigs were marked by NCBI BLAST to conﬁrm the sites within the closed genomes.

The complete genome sequence of Brucella canis GB1 was found to be 3,277,308 bp in size and have two circular chromosomes, chromosome I (2,106,982 bp) and chromosome II (1,170,326 bp). The number of contigs was 271, with an average contig length of 12.11 kb, coverage of 99.98%, sequencing depth of 477×, *N*_50_ value of 2,106,982 nucleotides (nt), and average G+C content of 57.2%. Meanwhile, the number of gaps was 23, and the total length of the gaps was 314,860 bp, which were ampliﬁed using primers designed by Primer Premier 5 and sequenced.

We then annotated the genome sequence. Open reading frames (ORFs) were predicted with GeneMarkS (7), rRNA was predicted by using RNAmmer 1.2 (8), and tRNA was identified with tRNAscan-SE 1.21 (9). The results showed that a total of 3,232 ORFs were predicted, including 3,104 protein-coding sequences, 55 tRNAs, 9 rRNAs, 4 noncoding RNAs (ncRNAs), 55 minisatellite DNAs, 18 microsatellite DNAs, and 55 tandem-repeat sequences. The most closely related genome was that of B. canis 2010009751.

### Data availability.

The complete genome sequence of B. canis GB1 was deposited in the NCBI GenBank under accession numbers CP027642 and CP027643. The raw reads of sequenced genomic DNA of B. canis GB1 were deposited in SRA under accession number PRJNA507342.
